# Intra-Specific Variation and Correlation of Functional Traits in *Cunninghamia lanceolata* at Different Stand Ages

**DOI:** 10.3390/plants14172675

**Published:** 2025-08-27

**Authors:** Jiejie Jiao, Chuping Wu, Honggang Sun, Liangjing Yao

**Affiliations:** 1 Zhejiang Academy of Forestry, Hangzhou 310023, China; jjjjust@163.com (J.J.); wcp1117@hotmail.com (C.W.); 2 Zhejiang Hangzhou Urban Forest Ecosystem Research Station, Hangzhou 310023, China; 3 Research Institute of Subtropical Forestry, Chinese Academy of Forestry, Hangzhou 311400, China; hogngangsun@caf.ac.cn

**Keywords:** functional traits, intra-specific variation, *Cunninghamia lanceolata*, trait trade-offs, ecological adaptation

## Abstract

Intra-specific variation in functional traits and their inter-relationships reflect how plants allocate resources, adapt, and evolve in response to environmental changes. This study investigated eight functional traits—leaf area (LA), specific leaf area (SLA), leaf dry matter content (LDMC), chlorophyll content (CHL), leaf nitrogen content (LNC), leaf phosphorus content (LPC), twig tissue density (TTD), and wood density (WD)—in *Cunninghamia lanceolata* plantations of three stand ages (15, 30, and 50 years), using a space-for-time substitution approach. We examined differences in trait values, intra-specific variation, and trait correlations across forest ages and diameter classes. The results showed that (1) Functional traits exhibited varying degrees of intra-specific variation, with LA having the highest coefficient of variation (21.66%) and LPC is lowest (9.31%). (2) Forest age had a stronger influence on trait variation than diameter class, with all traits differing significantly across ages, while only WD varied significantly among diameter classes. (3) PC1 (25.5%) and PC2 (19.4%) together explained approximately 44.9% of the total variation, with PC1 primarily reflecting functional trait changes driven by forest age. PCA results showed that LA and CHL tended to exhibit higher values in young forests, whereas SLA, LDMC, LPC, and LNC had relatively higher values in mature forests. This pattern suggests a shift in functional trait expression from resource acquisition to resource conservation strategies with increasing forest age. (4) Significant positive correlations between LNC and LPC, and negative correlations between SLA and LDMC, were observed in most groups, except in large-diameter trees at the over-mature stage. *C. lanceolata* adjusts trait combinations to enhance fitness across developmental stages. Juvenile trees adopt traits favoring efficient light and nutrient use to support rapid growth and competition. Middle-aged trees prioritize balanced water and nutrient use to maintain productivity and resist disturbances. Mature trees focus on sustained resource use and offspring protection to support ecosystem stability and regeneration. These findings reveal age-specific adaptive strategies and provide insights into the coordination and trade-offs among traits in response to environmental conditions.

## 1. Introduction

Species are the fundamental units of biology and ecology, and the interactions between plants and their environment lie at the core of plant functional ecology research [1,2,3]. Plant functional traits—key characteristics linked to survival, growth, and reproduction—mediate these interactions and influence both ecosystem function and vegetation responses to environmental change [4,5,6]. In the context of ongoing global climate change, trait-based ecological research methods have been extensively employed to predict species distribution ranges, phenology, and extinction risks associated with changing environmental conditions [7,8,9]. These methods also represent a crucial strategy for analyzing and objectively articulating the mechanisms through which plants adapt to external environmental changes [10].

It is well established that, to survive under resource-limited conditions, plants have evolved coordinated trait combinations that reflect trade-offs in resource allocation [11,12]. These trade-offs are foundational to classic ecological theories: Grime’s CSR (Competitive–Stress–Ruderal) framework posits that plants adjust traits to adapt to gradients of competition, environmental stress, and disturbance, with resource acquisition traits dominating in high-competition environments and conservation traits in stressful conditions [13,14]. Meanwhile, Tilman’s resource competition theory emphasizes that species differentiate their resource use efficiency to reduce niche overlap, which is manifested in dynamic trait adjustments across developmental stages [15]. These theories, alongside the widely recognized Leaf Economics Spectrum (LES) framework [16], provide a conceptual basis for understanding resource-use strategies. The LES illustrates global patterns in leaf trait coordination (e.g., trade-offs between specific leaf area and leaf dry matter content), while Grime and Tilman’s work extends this to broader life history strategies and community assembly [17,18]. However, many studies continue to focus on a single ecosystem function (e.g., productivity), overlooking potential trade-offs or synergies among functions, which may lead to biased conclusions [19]. To address this gap, our study integrates multiple functional indicators to explore how these theories apply to long-lived woody species like *C. lanceolata*.

In addition, early trait-based studies often relied on species-level means and overlooked intra-specific variation [20,21]. Intra-specific trait variation (ITV) refers to the range of trait values expressed by individuals within a species, resulting from genetic variation and phenotypic plasticity [22]. Notably, with advancements in research, ITV has been found to significantly contribute to overall trait variation and is crucial for assessing eco-evolutionary dynamics [23]. ITV buffers the potential decline in fitness caused by environmental changes and enables species to adapt rapidly, thereby enhancing the genetic diversity and evolutionary potential of populations [24,25]. This, in turn, shapes a species’ adaptive capacity and niche breadth [26,27]. Therefore, studying this variation within well-defined systems can provide key insights into plant adaptation mechanisms. However, the extent to which intra-specific trait variation conforms to such trade-offs remains underexplored, particularly in long-lived woody species under managed conditions, as its degree of variation is influenced by species type [28], age and developmental stage [29], and habitat context [30]. Forest management practices have demonstrated that the developmental stages of forests are frequently associated with alterations in resource availability, microclimate, and soil conditions, which in turn induce transformations in plant morphology and physiological characteristics [31]. Diameter at breast height (DBH) serves as a crucial indicator for assessing individual competitiveness and growth performance [32,33]. Consequently, investigating how functional traits (e.g., wood density and specific leaf area) vary with forest age and DBH can help uncover life history strategies and guide forest management.

*C. lanceolata*, a fast-growing conifer widely cultivated in subtropical China, represents 17% of China’s plantation area, and plays a crucial role in timber production and forest carbon sequestration in the country [34]. Variation in its functional traits can affect growth, stress resistance, and carbon sequestration potential. For instance, traits influencing photosynthesis and respiration directly relate to its carbon balance [5,20], while others contribute to pest resistance and environmental stress tolerance [4,25]. Understanding this variation can inform targeted silvicultural practices, such as thinning and trait-based selection, to enhance stand productivity, resilience, and carbon sink function [25]. However, current research on *C. lanceolata* plantation management strategies primarily focuses on mixed-species configurations, superior provenance selection, and close-to-nature management. Additionally, while existing studies have demonstrated significant intra-specific variability in the functional traits of *C. lanceolata* leaves, investigations have largely been limited to comparisons among different provenances [35,36,37,38,39,40], with few systematic examinations of how these functional traits vary across different age classes. To better understand how *C. lanceolata* adapts to environmental changes across developmental stages, this study aimed to analyze the intra-specific variation and coordination of key functional traits across different stand ages and diameter classes. We hypothesized that (1) functional traits would exhibit significant intra-specific variation, with leaf traits showing greater variability than stem traits; (2) forest age would have a stronger influence on trait expression than tree size (DBH class), reflecting ontogenetic shifts in resource-use strategies; and (3) trait coordination patterns would vary with stand development, indicating age-specific trade-offs between resource acquisition and conservation strategies. By testing these hypotheses, our study provides a mechanistic understanding of how functional traits underpin adaptation and resource allocation in *C. lanceolata*, offering valuable implications for plantation management and sustainable forest development.

## 2. Results

### 2.1. Intra-Specific Variation in Functional Traits

The coefficients of variation (CV) for the eight functional traits of *C. lanceolata* ranged from 9.31% to 21.66%. LA and SLA exhibited the highest variability (CV > 20%), indicating pronounced plasticity, while twig tissue density (TTD: 11.10%), leaf nitrogen content (LNC: 12.22%), and leaf phosphorus content (LPC: 9.31%) showed relatively low variation. Among different forest ages, 30-year-old trees exhibited the greatest variability in leaf nutrient traits (LNC and LPC), whereas in 50-year-old trees, higher variation was observed in CHL, LDMC, TTD, and WD. Across diameter classes, WD consistently decreased with increasing diameter, while the variation trends of other traits were inconsistent (Table 1).

### 2.2. Influence of Forest Age and Diameter Class on Functional Traits

**Figure 1:**
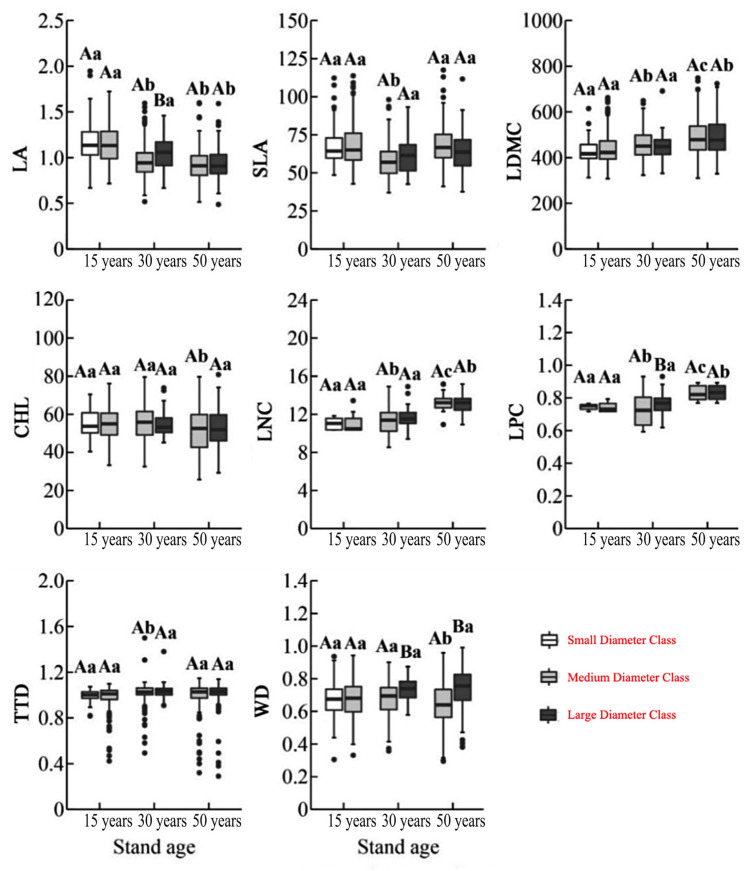
Variation in functional traits of *C. lanceolata* across different stand ages and diameter classes. Different uppercase letters indicate significant differences (*p* < 0.05) in functional traits among diameter classes (small, medium, large), while different lowercase letters indicate significant differences (*p* < 0.05) among stand ages (15, 30, and 50 years). Functional traits include Leaf Area (LA), Specific Leaf Area (SLA), Leaf Dry Matter Content (LDMC), Chlorophyll Content (CHL), Leaf Nitrogen Content (LNC), Leaf Phosphorus Content (LPC), Twig Tissue Density (TTD), and Wood Density (WD).

Two-way ANOVA revealed that forest age had a significant effect on all eight functional traits (*p* < 0.01), while diameter class significantly influenced only WD (*p* < 0.01). The interaction between forest age and diameter class significantly affected LNC and LPC (*p* < 0.001), suggesting age as the dominant factor shaping trait variation. Among forest ages, 15-year-old trees exhibited the largest LA, whereas 30-year-old individuals had the highest TTD and the lowest SLA. In contrast, 50-year-old trees showed the lowest CHL and highest values for LDMC, LNC, and LPC. With increasing diameter, LA decreased, while LDMC, LNC, and LPC generally increased. WD was significantly higher in large-diameter trees compared to medium-diameter trees; other traits showed no consistent pattern (Figure 1).

### 2.3. Principal Component Analysis (PCA) Under Standardized Functional Trait Data

**Figure 2:**
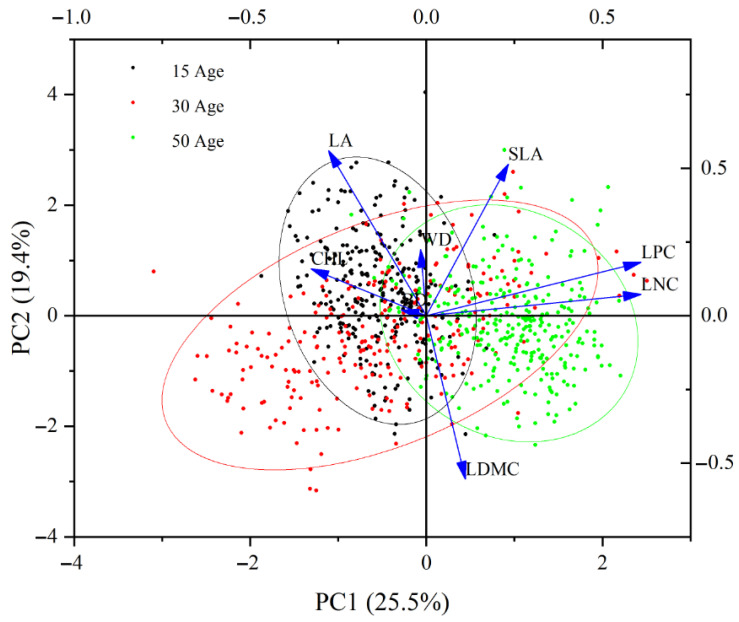
Principal component analysis (PCA) of functional traits in *C. lanceolata* of different stand ages.

PCA of standardized functional trait data across different ages of *C. lanceolata* forests indicated that PC1 (25.5%) and PC2 (19.4%) collectively accounted for approximately 44.9% of the variation in the data (Figure 2). PC1 primarily reflects the changes in functional traits induced by variations in forest age. The distribution of young forests (15a) was skewed towards the left side of the plot, while middle-aged forests (30a) were centrally located, and mature forests (50a) are skewed towards the right side. LA and CHL were more strongly associated with young forests, indicating that these traits were more pronounced in younger stands. Conversely, SLA, LDMC, LPC, and LNC were associated with mature forests, suggesting that these traits become more prominent in older stands. Therefore, there was a distinct shift in functional traits from young to mature forests, characterized by the transition from high SLA/CHL to high LDMC/LNC.

### 2.4. Trait Correlations Across Ages and Diameter Class Classes

**Figure 3:**
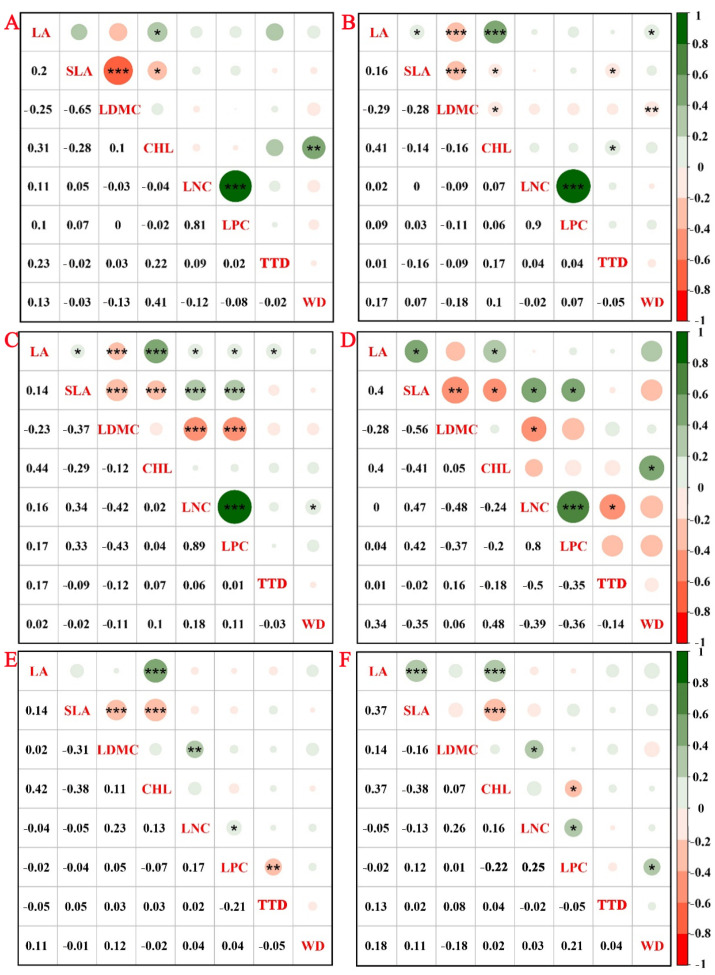
Correlation of functional traits in *C. lanceolata* across different stand ages (15, 30, and 50 years) and diameter classes. Note: 15-year-old stands with small (**A**) and medium (**B**) diameter classes, 30-year-old stands with small (**C**) and medium (**D**) diameter classes, and 50-year-old stands with medium (**E**) and large (**F**) diameter classes. * Indicates a significant correlation (*p* < 0.05), ** indicates a highly significant correlation (*p* < 0.01), and *** indicates the most significant correlation (*p* < 0.001). The correlation coefficients are shown alongside their respective *p*-values.

Pearson correlation analysis showed that LA was positively correlated with Chl and negatively with SLA across all forest ages and diameter classes. LNC was positively correlated with LPC, indicating coordinated nutrient allocation. Strongest trait inter-correlations were observed in 30-year-old stands. With increasing diameter, the positive correlation between LA and SLA became more pronounced. In 50-year-old trees, the previously negative correlation between SLA and LDMC diminished and became non-significant. Similarly, the relationship between LDMC and LNC shifted from significantly negative in younger stands to significantly positive in older stands. In large-diameter trees, the expected trade-off between SLA and LDMC was absent (Figure 3).

## 3. Discussion

Functional traits in plants encompass morphological, physiological, and phenological characteristics that indirectly influence plant fitness by affecting individual performance and interactions with the environment [5]. However, in the face of intense climate change, functional traits at different growth stages of the same species may exhibit significant and varied intra-specific variations [41,42,43]. Furthermore, phenotypic plasticity can differ at the intra-specific level in relation to individual fitness, manifesting as adaptive, maladaptive, or non-adaptive responses [44]. Intra-specific variation is defined as the phenotypic or genetic differences within a species, reflecting the responses of plants to their environment [41]. Therefore, assessing the intra-specific variation patterns of plant functional traits and their manifestation across different growth stages can provide new insights into understanding the survival and adaptation strategies of plants.

Our findings confirmed that significant intra-specific variation exists among key leaf and stem traits in *C. lanceolata*, particularly for leaf area (LA) and specific leaf area (SLA). These two traits exhibited the highest coefficients of variation, with SLA exceeding 20%, indicating strong trait plasticity during early growth stages. As is well known, among the numerous plant trait characteristics, leaf traits—such as leaf area and specific leaf area—and their intra-specific variation can elucidate plant resource acquisition strategies and functional optimization strategies across diverse spatial and temporal scales [30,42]. Additional studies have shown that as trees mature and canopy closure increases, their leaf area tends to decrease. Most resources are allocated more towards structural growth and reproduction, such as the development of stems and roots [20,27,43], or by adjusting leaf characteristics to optimize photosynthesis, rather than merely increasing leaf area (etc., the plant light competition model) [26,45]. In this study, LA and SLA exhibited the highest variation among the functional traits of *C. lanceolata*, indicating that these traits are significantly driven by forest age factors, which is consistent with the findings of Wang et al. [46]. However, leaf chemical traits exhibited more stability across stand ages, in line with ecological stoichiometry, where plants maintain a consistent chemical composition despite environmental fluctuations [43]. Contrasting findings by Xu et al. [39] suggest that intra-specific variation in leaf chemical traits might be more pronounced than in structural traits, potentially due to differences in soil nutrient availability across various forest ages [47]. Additionally, branch traits exhibited conservative responses to environmental changes, likely due to slower branch growth and delayed responses to habitat shifts [20].

Existing research has demonstrated that throughout the life cycle of plants, their functional traits are influenced by environmental factors, including resource availability, biological competition, and soil nutrient levels [3,48]. Notably, these factors evolve as the stand age increases [49]. In this study, the impact of diameter class differences on functional traits becomes less pronounced with radial growth, indicating that stand age plays a more significant role than diameter class in determining functional trait variation [50]. During early growth, *C. lanceolata* increases leaf area and SLA to maximize photosynthetic efficiency, a physiological strategy that facilitates rapid growth during its fast-growing period [51]. As the tree matures, leaf area gradually decreases while the dry matter content of the leaves increases to reduce water loss and transfer more resources (such as energy and nutrients) to the tissue components to promote structural growth [52]. In addition, nitrogen and phosphorus are key substances for plants to complete photosynthesis and enhance photosynthetic efficiency [53,54]. Notably, leaf nitrogen (LNC) and phosphorus content (LPC) also demonstrated age-related variation, with LNC increasing and LPC showing a U-shaped trend. This suggests dynamic nutrient allocation during stand development, likely influenced by litter decomposition, soil nutrient availability, and reproductive demands [47,53,54,55]. Furthermore, the observed increase in wood density (WD) in large-diameter trees aligns with mechanical support needs during late-stage growth [50,56]. Beyond trait means, our study also revealed important patterns of trait coordination. In contrast to focusing on a single trait, plants typically operate as an integrated system, and the interplay and trade-offs among various plant functional traits can effectively illustrate how plants respond to external environmental conditions [57,58]. In this study, the functional traits of *C. lanceolata* exhibited consistent correlations across different stand ages and diameter classes. The positive relationship between leaf area and chlorophyll content is likely due to their combined influence on photosynthesis, while the correlation between leaf N and P content reflects their shared absorption and transport mechanisms within plants [45,53]. Strong positive correlations were consistently observed between LA and chlorophyll content (CHL), and between LNC and LPC, indicating coordinated nutrient and photosynthetic trait responses. Meanwhile, a significant negative correlation between SLA and LDMC represents a trade-off between photosynthetic efficiency and structural investment, a fundamental axis of the global Leaf Economics Spectrum (LES) [59,60]. Crucially, we identified changes in trait correlation strength and direction with age, emphasizing the dynamic nature of trait integration. For example, the relationship between LDMC and LNC shifted from negative in younger stands to positive in older stands, potentially due to altered growth rates and nutrient cycling as trees age [61,62]. This highlights the plastic coordination of functional traits in response to long-term environmental pressures and stand development. Lastly, the 30-year-old stands exhibited the strongest overall trait correlations, suggesting enhanced functional integration and adaptability at mid-successional stages [61]. This could represent a critical phase where *C. lanceolata* achieves peak ecological fitness through optimized trait combinations. As stand age increased, we observed a clear ontogenetic shift from resource acquisition to resource conservation. Specifically, juvenile trees in 15-year-old stands adopted a “fast-return” strategy characterized by high SLA and low leaf dry matter content (LDMC), enabling rapid biomass accumulation. In contrast, 30-year-old stands exhibited reduced SLA and increased LDMC, reflecting a “slow-return” strategy with enhanced investment in structural and stress-resistance traits [16,63]. Interestingly, in 50-year-old stands, large-diameter trees showed a novel trait combination of high SLA and high LDMC, suggesting a mixed strategy to balance efficient photosynthesis with structural stability, an adaptive optimization not commonly reported in conifers [64,65]. These findings suggest that *C. lanceolata* adjusts its functional traits in response to changes in resource availability and environmental pressures, forming an optimal trait combination through long-term adaptation. Similar patterns of functional trait variation with age and environmental factors have been observed in other conifer species, such as *Pinus taeda* and *Picea glauca*, highlighting shared adaptive strategies across conifer species [66]. In summary, our study underscores that intra-specific variation and dynamic trait coordination are central to the ecological strategy of *C. lanceolata*. The species modulates its traits across stand development stages to adapt to shifting environmental and physiological demands. These findings provide a nuanced understanding of trait-based adaptation in conifers and offer valuable insights for sustainable forest management under changing environmental conditions. Future research should integrate environmental variables (e.g., soil nutrients, microclimate, stand density) to further clarify the drivers of trait variation and their implications for productivity, resilience, and ecosystem service provisioning in *C. lanceolata* plantations.

## 4. Materials and Methods

### 4.1. Study Area

This study was conducted in Huangtan Forest Farm, Pan’an County, Zhejiang Province (120°16′–120°26′ E, 28°42′–28°52′ N), covering 847.4 ha with a forest coverage rate of 96.78%. The region is characterized by mid- to low-mountainous terrain with elevations averaging 900 m and slopes ranging from 15° to 35°. It has a subtropical monsoon climate, with distinct seasonal variations, warm and humid conditions, as well as abundant moisture, light, and heat resources [66]. According to the World Soil Resources Reference Base (WRB) classification [67], the dominant soil type is yellow soil. Detailed information of the forest stands is shown in Table 2.

### 4.2. Plot Design and Sampling Strategy

**Figure 4:**
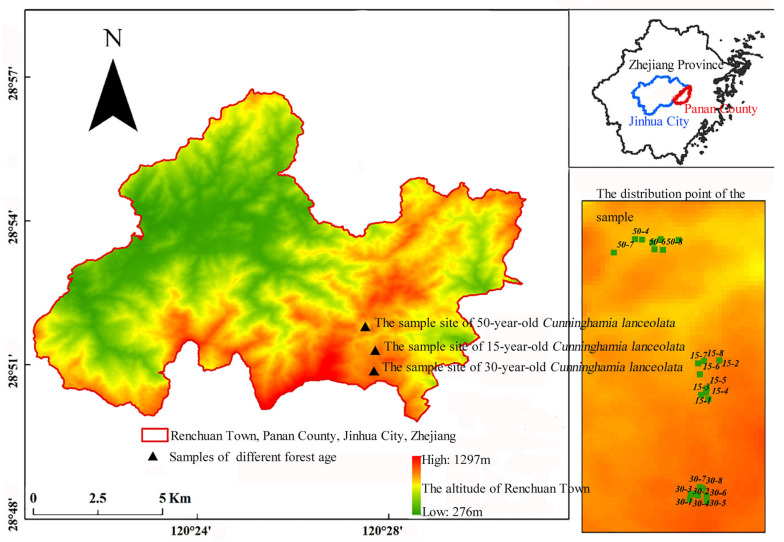
Overview of the sampling plots.

In May–June 2022, pure *C. lanceolata* plantations of 15, 30, and 50 years of age (planted in 2008, 1993, and 1973, respectively) were selected under similar site conditions. All stands originated from the same seed source and were established using two-year-old seedlings at a density of 3000 trees ha^−1^ (Figure 4). Final thinning was conducted in 2015, and no natural regeneration of broad-leaved species was observed. Plots were weeded regularly in summer, and no irrigation or fertilization was applied.

For each age class, eight permanent plots (20 × 20 m) were established following the CTFS protocol [68], totaling 24 plots. Each plot was subdivided into 16 subplots (5 × 5 m) for systematic sampling. All *C. lanceolata* individuals with DBH ≥ 5.0 cm were measured. Based on actual plot survey data combined with the exclusion method using upper limits, the DBH (diameter at breast height) of *C. lanceolata* plantations at different stand ages was classified into three diameter classes: small diameter class: 5 cm ≤ DBH < 13 cm, medium diameter class: 13 cm ≤ DBH < 25 cm, large diameter class: DBH ≥ 25 cm [69].

### 4.3. Trait Sampling and Measurement

From July to September 2022, 15 individuals per diameter class were randomly selected in each plot. If fewer than 15 trees were present, all individuals were sampled. For each tree, branches were sampled from the sun-exposed side of the canopy. Five healthy, fully expanded leaves were collected, along with ~10 cm long non-current-year twigs (1–2 cm diameter), and one wood core at breast height using an increment borer with an internal diameter of 5.15 mm (Haglof, Sweden). Samples were kept moist and transported to the laboratory for immediate processing.

Eight functional traits were measured. Leaf traits: leaf area (LA, cm^2^), specific leaf area (SLA, cm^2^·g^−1^), leaf dry matter content (LDMC, mg·g^−1^), chlorophyll content (CHL, SPAD), leaf nitrogen (LNC, mg·g^−1^), and phosphorus content (LPC, mg·g^−1^). Stem traits: twig tissue density (TTD, g·cm^−3^), and wood density (WD, g·cm^−3^). Trait measurements followed standardized protocols in the “Plant Trait Measurement Manual” [44]. LA was measured using a leaf area meter (Li-3000A, LI-COR Biosciences, USA). SLA was calculated as the ratio of leaf area to oven-dried mass (60 °C, 48 h). LDMC was the ratio of oven-dried mass to fresh mass. CHL was estimated non-destructively with a SPAD-502 meter (Konica Minolta, Japan). LNC was determined by dry combustion using an elemental analyzer, while LPC was measured via spectrophotometry following acid digestion. TTD and WD were determined as dry mass divided by volume, with twig and wood volume calculated via water displacement. In total, 863 samples were collected for each trait across age and diameter classes, allowing for a comprehensive assessment of intra-specific trait variation.

### 4.4. Statistical Analysis

Trait data were first tested for normality using the Shapiro–Wilk test. Two-way ANOVA was applied to assess the effects of forest age, diameter class, and their interaction. One-way ANOVA and post hoc comparisons using the Least Significant Difference (LSD) test were then conducted to examine variation across age and diameter classes. Intra-specific variation was quantified using the coefficient of variation (CV = SD/mean × 100%). PCA of various functional traits was conducted using the FactoMineR package (version 4.3.3). Pearson correlation analysis was performed to evaluate relationships among traits. All statistical analyses and visualizations were conducted using R software (version 4.0.3). Statistical significance was defined as follows: *p* < 0.05 (significant), *p* < 0.01 (highly significant), and *p* < 0.001 (very highly significant).

## 5. Conclusions

This study revealed marked variations in the functional traits of *Cunninghamia lanceolata* across stand ages, underscoring divergent ecological adaptation strategies. Younger trees (15 years) adopted a “fast-return” strategy with high SLA and low LDMC, while middle-aged trees (30 years) exhibited a “slow-return” strategy characterized by higher LDMC and lower SLA. In older stands (50 years), trait variation was evident across diameter classes, suggesting a complex allocation strategy, particularly in larger individuals with simultaneously high SLA, LDMC, and LNC. These findings highlight ontogenetic shifts and within-stand heterogeneity in adaptive strategies. Our results align with Grime’s CSR framework and Tilman’s resource competition theory, providing empirical support from a long-lived conifer. The ontogenetic shift from resource acquisition to conservation traits corresponds to Grime’s stress-tolerance strategy in mature stands, while trait differentiation across diameter classes reflects Tilman’s niche partitioning via resource use efficiency. Future research should focus on disentangling the mechanisms by which soil properties and stand structure interact with tree ontogeny to shape functional traits. Comparative studies across regions and site conditions would further enhance our understanding of the ecological plasticity and adaptive strategies of *C. lanceolata*, thereby extending classic trait-based theories into the context of managed forest ecosystems.

## Figures and Tables

**Table 1:** Functional trait characteristics and coefficients of variation (CV) of *C. lanceolata* across different forest ages and DBH classes.

Stand Age	DBH Class	Statistics	Functional Traits							
			LA	SLA	LDMC	CHL	LNC	LPC	TTD	WD
15 years	Small Diameter Class (5 cm ≤ DBH < 13 cm)	Mean ± Se	1.18 ± 0.24 ^A^	69.48 ± 15.30 ^A^	424.02 ± 61.42 ^A^	54.02 ± 7.21 ^A^	11.01 ± 0.61 ^A^	0.74 ± 0.02 ^A^	0.99 ± 0.05 ^A^	0.67 ± 0.11 ^A^
		*CV*	20.59%	22.02%	14.48%	13.35%	5.53%	2.36%	5.36%	16.74%
	Medium Diameter Class (13 cm ≤ DBH < 25 cm)	Mean ± Se	1.15 ± 0.22 ^Aa^	68.07 ± 14.27 ^Aa^	439.74 ± 66.91 ^Aa^	54.16 ± 7.67 ^Aa^	10.88 ± 0.64 ^Aa^	0.73 ± 0.02 ^Aa^	0.98 ± 0.10 ^Aa^	0.67 ± 0.11 ^Aa^
		*CV*	19.22%	20.96%	15.21%	14.16%	5.89%	2.82%	10.09%	16.62%
30 years	Medium Diameter Class	Mean ± Se	0.97 ± 0.17 ^Ab^	57.63 ± 10.84 ^Ab^	456.92 ± 61.25 ^Ab^	55.24 ± 8.88 ^Aa^	11.24 ± 1.51 ^Ab^	0.72 ± 0.09 ^Ab^	1.02 ± 0.09 ^Ab^	0.67 ± 0.10 ^Aa^
		*CV*	18.11%	18.81%	13.40%	16.07%	13.41%	12.64%	9.11%	15.58%
	Large Diameter Class (DBH ≥ 25 cm)	Mean ± Se	1.07 ± 0.19 ^Ba^	61.87 ± 12.80 ^Aa^	449.50 ± 69.64 ^Aa^	54.53 ± 7.53 ^Aa^	11.79 ± 1.16 ^Aa^	0.76 ± 0.08 ^Ba^	1.04 ± 0.09 ^Aa^	0.74 ± 0.08 ^Ba^
		*CV*	17.71%	20.70%	15.49%	13.81%	9.87%	9.87%	8.46%	10.31%
50 years	Medium Diameter Class	Mean ± Se	0.93 ± 0.18 ^Ab^	68.87 ± 13.41 ^Aa^	488.94 ± 81.73 ^Ac^	50.57 ± 11.78 ^Ab^	13.23 ± 0.82 ^Ac^	0.82 ± 0.04 ^Ac^	0.99 ± 0.13 ^Aa^	0.64 ± 0.13 ^Ab^
		*CV*	19.67%	19.47%	16.72%	23.29%	6.21%	5.21%	13.07%	20.78%
	Large Diameter Class	Mean ± Se	0.94 ± 0.19 ^Ab^	65.36 ± 15.11 ^Aa^	494.69 ± 82.50 ^Ab^	51.13 ± 11.67 ^Aa^	13.12 ± 0.94 ^Ab^	0.82 ± 0.04 ^Ab^	1.00 ± 0.14 ^Aa^	0.74 ± 0.13 ^Ba^
		*CV*	20.20%	23.11%	16.68%	22.82%	7.14%	5.21%	14.47%	17.25%
Overall coefficient of variation *CV*			21.66%	21.19%	16.05%	18.12%	12.22%	9.31%	11.10%	17.88%

Different uppercase letters indicate significant differences (*p* < 0.05) among diameter classes (small, medium, and large), while different lowercase letters indicate significant differences (*p* < 0.05) among stand ages (15, 30, and 50 years). Values are presented as means ± standard error (Se). The coefficient of variation (*CV*) is expressed as a percentage (%).

**Table 2:** Information on *C. lanceolata* plantation.

Study Area	Characteristics of Stand		Information of Climate		Characteristics of Soil (0–30 cm)	
Huangtan Forest Farm, Pan’an County, Zhejiang Province, China	ST	Artificial pure forest	CT	Subtropical monsoon climate	ST	Yellow soil
					pH	7.3
	AT	1973, 1993, and 2008	MAP	1650 mm	C	34.89 g·kg^−1^
			MAT	15.0 °C	N	1.16 g·kg^−1^
	PD	3000 trees ha^−1^ (2-year-old plantings)	FFP	193 d	P	0.71 g·kg^−1^
			AAT	4894.5 °C	K	11.73 g·kg^−1^
	Main vegetation under the forest		*Aster ageratoides*, *Saxifraga stolonifera*, *Asparagus cochinchinensis*, *Ligustrum quihoui*, *Miscanthus sinensis*, *Lindera aggregata*, and *Akebia trifoliata* et al.			

ST: Stand type; AT: Afforestation time; PD: Planting density; CT: Climate type; MAP: Mean annual precipitation; MAT: Mean annual temperature; FFP: Frost-free period; AAT: Annual average accumulated temperature; ST: Soil type; C: Soil organic matter content; N: Soil total nitrogen content; P: Soil total phosphate content; K: Soil total potassium.

## Data Availability

The data presented in this study are available upon request from the corresponding author. The data are not publicly available due to ethical reasons.

## References

[1] Hohenegger J. (2014). Species as the basic units in evolution and biodiversity: Recognition of species in the recent and ceological past as exemplified by larger foraminifera. Gondwana Res..

[2] Buckley Y.M., Austin A., Bardgett R., Catford J.A., Hector A., Iler A., Mariotte P. (2024). The plant ecology of nature-based solutions for people, biodiversity and climate. J. Ecol..

[3] Anderegg L.D.L. (2023). Why can’t we predict traits from the environment?. New Phytol..

[4] Díaz S., Cabido M. (2001). Vive la différence: Plant functional diversity ma tters to ecosystem processes. Trends Ecol. Evol..

[5] Funk J.L., Larson J.E., Ames G.M., Butterfield B.J., Cavender-Bares J., Firn J., Laughlin D.C., Sutton-Grier A.E., Williams L., Wright J. (2017). Revisiting the Holy Grail: Using plant functional traits to understand ecological processes. Biol. Rev..

[6] Falster D.S., Duursma R.A., FitzJohn R.G. (2018). How functional traits influence plant growth and shade tolerance across the life cycle. Proc. Natl. Acad. Sci. USA.

[7] Aguirre-Gutiérrez J., Díaz S., Rifai S.W., Corral-Rivas J.J., Nava-Miranda M.G., González-M R., Hurtado-M A.B., Revilla N.S., Vilanova E., Almeida E. (2025). Tropical forests in the Americas are changing too slowly to track climate change. Science.

[8] Gremer J.R. (2023). Looking to the past to understand the future: Linking evolutionary modes of response with functional and life history traits in variable environments. New Phytol..

[9] He P., Lian J., Ye Q., Liu H., Zheng Y., Yu K., Zhu S., Li R., Yin D., Ye W. (2022). How do functional traits influence tree demographic properties in a subtropical monsoon forest?. Funct. Ecol..

[10] Duan X., Jia Z., Li J., Wu S. (2022). The influencing factors of leaf functional traits variation of *Pinus densiflora* Sieb. et Zucc. Glob. Ecol. Conserv..

[11] Pan L., Wang T., Gavrikov V.L., Guo X., Mu L., Tang Z. (2025). Trade-off strategies between growth and defense of spring ephemeral plants in early sprin. Front. Plant Sci..

[12] Yang Y., Wang H., Harrison S.P., Prentice I.C., Wright I.J., Peng C., Lin G. (2019). Quantifying leaf-trait covariation and its controls across climates and biomes. New Phytol..

[13] Grime J.P. (2006). Trait convergence and trait divergence in herbaceous plant communities: Mechanisms and consequences. J. Veg. Sci..

[14] Grime J.P. (2006). Plant Strategies, Vegetation Processes, and Ecosystem Properties.

[15] Tilman D. (1982). Resource Competition and Community Structure.

[16] Wright I.J., Reich P.B., Westoby M., Ackerly D.D., Baruch Z., Bongers F., Cavender-Bares J., Chapin T., Cornelissen J.H.C., Diemer M. (2004). The worldwide leaf economics spectrum. Nature.

[17] He J.S., Wang Z., Wang X., Schmid B., Zuo W., Zhou M., Zheng C., Wang M., Fang J. (2006). A test of the generality of leaf trait relationships on the Tibetan Plateau. New Phytol..

[18] Ji W., LaZerte S.E., Waterway M.J., Lechowicz M.J. (2020). Functional ecology of congeneric variation in the leaf economics spectrum. New Phytol..

[19] Gross N., Bagousse-Pinguet Y.L., Liancourt P., Berdugo M., Gotelli N.J., Maestre F.T. (2017). Functional trait diversity maximizes ecosystem multifunctionality. Nat. Ecol. Evol..

[20] Heilmeier H. (2019). Functional traits explaining plant responses to past and future climate changes. Flora.

[21] Palacio F.X., Fernández G.J., Ordano M. (2019). Does accounting for within-individual trait variation matter for measuring functional diversity?. Ecol. Indic..

[22] He Y., Junker R.R., Xiao J., Lasky J.R., Cao M., Asefa M., Swenson N.G., Xu G., Yang J., Sedio B.E. (2025). Genetic and environmental drivers of intraspecific variation in foliar metabolites in a tropical tree community. New Phytol..

[23] Tusifujiang Y., Zhang X., Gong L. (2021). The relative contribution of intraspecific variation and species turnover to the community-level foliar stoichiometric characteristics in different soil moisture and salinity habitats. PLoS ONE.

[24] Liu H., Yin D., He P., Cadotte M.W., Ye Q. (2024). Linking plant functional traits to biodiversity under environmental change. Biol. Divers..

[25] Laforest-Lapointe I., Martínez-Vilalta J., Retana J. (2014). Intraspecific variability in functional traits matters: Case study of Scots pine. Oecologia.

[26] Yao L., Wu C., Wang Z., Jiang B. (2024). Alpha and beta diversity of functional traits in subtropical evergreen broad-leaved secondary forest communities. Front. Plant Sci..

[27] Albert C.H., Grassein F., Schurr F.M., Vieilledent G., Violle C. (2011). When and how should intraspecific variability be considered in trait-based plant ecology?. Perspect. Plant Ecol. Evol. Syst..

[28] Zhang B., Li X., Chen H., Deng M., Xiao H., Dong S., Scheu S., Wang S. (2025). Adult body mass influences multi-element stoichiometry in ground beetles. Soil. Biol. Biochem..

[29] Cope O.L., Burkle L.A., Croy J.R., Mooney K.A., Yang L.H., Wetzel W.C. (2022). The role of timing in intraspecific trait ecology. Trends Ecol. Evol..

[30] Puglielli G., Bricca A., Chelli S., Petruzzellis F., Acosta A.T.R., Bacaro G., Bacaro E., Bernardo L., Bonari G., Bolpagni R. (2024). Intraspecific variability of leaf form and function across habitat types. Ecol. Lett..

[31] Oktavia D., Park J.W., Jin G. (2022). Life stages and habitat types alter the relationships of tree growth with leaf traits and soils in an old-growth temperate forest. Flora.

[32] Chen C., Wen Y., He B., Yang Y., Han X., Sun T., Lu X. (2023). Environmental factors driving the succession and differentiation of ecological strategy spectrum in tropical lowland rain forest. Ecol. Indic..

[33] Qin Y., Wu B., Lei X., Feng L. (2023). Prediction of tree crown width in natural mixed forests using deeplearning algorithm. For. Ecosyst..

[34] Jia L., Jiang Q., Sun J., Robinson D., Yang Z., Yao X., Wang X., Dai X., Chen T., Wu D. (2024). Contrasting depth-related fine root plastic responses to soil warming in a subtropical Chinese fir plantation. J. Ecol..

[35] Hu Y., Jiang Y., Chhin S., Liu N., Pang H., Zhang J., Zhu G., Zhang X. (2025). Alleviating monoculture-induced soil degradation in Chinese fir plantations in southern China: Optimizing understory mixtures balances stoichiometry and microbial diversity. Ind. Crops Prod..

[36] Wang H., Duan A., Zhang J. (2025). Intraspecific responses to climate change in *Cunninghamia lanceolata* (Lamb.) Hook.: Local may not be the best. For. Ecol. Manag..

[37] Wan Z., Liu N., Liu C., Zhang M., Gao C., Yang L., Yao L., Zhang X. (2025). Comparison of growth strategies and biomass allocation in Chinese fir provenances from the subtropical region of China. Forests.

[38] Lei J., Wu H., Li X., Guo W., Duan A., Zhang J. (2023). Response of rhizosphere bacterial communities to near-natural forest management and tree species within Chinese fir plantations. Microbiol. Spectr..

[39] Xu R., Cheng S., Zhou J., Tigabu M., Ma X., Li M. (2023). Intraspecific variations in leaf functional traits of *Cunninghamia lanceolata* provenances. BMC Plant Biol..

[40] Jian M.P., Yang J.Y. (2024). Unveiling the adaptation strategies of woody plants in remnant forest patches to spatiotemporal urban expansion through leaf trait networks. For. Ecosyst..

[41] Li Y., Mo Y.X., Cui H.L., Zhang Y., Dossa G.G., Tan Z., Song L. (2024). Intraspecific plasticity and co-variation of leaf traits facilitate *Ficus tinctoria* to acclimate hemiepiphytic and terrestrial habitats. Tree Physiol..

[42] Lecerf A., Chauvet E. (2008). Intraspecificvariability in leaf traits strongly affects alder leaf decomposition in a stream. Basic Appl. Ecol..

[43] Lu J., Zhao X., Wang S., Feng S., Ning Z., Wang R., Chen X., Zhao H., Chen M. (2023). Untangling the influence of abiotic and biotic factors on leaf C, N, and P stoichiometry along a desert-grassland transition zone in northern China. Sci. Total Environ..

[44] Messier J., Lechowicz M.J., McGill B.J., Violle C., Enquist B.J. (2017). Interspecific integration of trait dimensions at local scales: The plant phenotype as an integrated network. J. Ecol..

[45] Kuebbing S.E., Bradford M.A. (2019). The potential for mass ratio and trait divergence effects to explain idiosyncratic impacts of non-native invasive plants on carbon mineralization of decomposing leaf litter. Funct. Ecol..

[46] Zheng S., Yu M., Webber B.L., Didham R.K. (2024). Intraspecific leaf trait variation mediates edge effects on litter decomposition rate in fragmented forests. Ecology.

[47] Osman K.T. (2013). Physical Properties of Forest Soils. Forest Soils.

[48] Su X., Zheng G., Chen H.Y.H. (2022). Understory diversity are driven by resource availability rather thanresource heterogeneity in subtropical forests. For. Ecol. Manag..

[49] Lambers H., Oliveira R.S. (2019). Biotic Influences: Interactions Among Plants. Plant Physiological Ecology.

[50] Iida Y., Swenson N.G. (2020). Towards linking species traits to demography and assembly in diverse tree communities: Revisiting the importance of size and allocation. Ecol. Res..

[51] Engbersen N., Stefan L., Brooker R.W., Schöb C. (2022). Using plant traits to understand the contribution of biodiversity effects to annual crop community productivity. Ecol. Appl..

[52] Zhu L., Sun J., Yao X., Wang X., Huang J., Xiong D., Chen G. (2022). Fine root nutrient foraging ability in relation to carbon availability along achronosequence of Chinese fir plantations. For. Ecol. Manag..

[53] Mu X., Chen Y. (2021). The physiological response of photosynthesis to nitrogen deficiency. Plant Physiol. Biochem..

[54] Qi J., Ma K., Zhang Y. (2008). Comparisons on leaf traits of *Quercus liaotungensis* Koidz. on different slope positions in Dongling Moutain of Beijing. Acta Ecol. Sin..

[55] Wu A., Hu X., Wang F., Guo C., Wang H., Chen F.S. (2021). Nitrogen deposition and phosphorus addition alter mobility of trace elements in subtropical forests in China. Sci. Total Environ..

[56] Chave J., Coomes D., Jansen S., Lewis S.L., Swenson N.G., Zanne A.E. (2009). Towards a worldwide wood economics spectrum. Ecol. Lett..

[57] He N., Li Y., Liu C., Xu L., Li M., Zhang J., He J., Tang Z., Han X., Ye Q. (2020). Plant trait networks: Improved resolution of the dimensionality of adaptation. Trends Ecol. Evol..

[58] Rao Q.Y., Chen J.F., Chou Q.C., Ren W., Cao T., Zhang M., Xiao H., Liu Z., Chen J., Su H. (2023). Linking trait network parameters with plant growth across light gradients and seasons. Funct. Ecol..

[59] Roche P., Díaz-Burlinson N., Gachet S. (2004). Congruency analysis of species ranking based on leaf traits: Which traits are the more reliable?. Plant Ecol..

[60] Khan A., Yan L., Hasan M.M., Wang W., Xu K., Zou G., Liu X.D., Fang X.W. (2022). Leaf traits and leaf nitrogen shift photosynthesis adaptive strategies among functional groups and diverse biomes. Ecol. Indic..

[61] Díaz S., Purvis A., Cornelissen J.H.C., Mace G.M., Donoghue M.J., Ewers R.M., Jordano P., Pearse W.D. (2013). Functional traits, the phylogeny of function, and ecosystem service vulnerability. Ecol. Evol..

[62] Huang Z., Liu Q., An B., Wu X., Sun L., Wu P., Liu B., Ma X. (2021). Effects of planting density on morphological and photosynthetic characteristics of leaves in different positions on *Cunninghamia lanceolata* saplings. Forests.

[63] Cornelissen J.H.C., Lavorel S., Garnier E., Díaz S., Buchmann N., Gurvich D.E., Reich P.B., Steege H.ter., Morgan H.D., van der Heijden M.G.A. (2003). A handbook of protocols for standardised and easy measurement of plant functional traits worldwide. Aust. J. Bot..

[64] Westoby M., Falster D.S., Moles A.T., Vesk P.A., Wright I.J. (2002). Plant ecological strategies: Some leading dimensions of variation between species. Annu. Rev. Ecol. Syst..

[65] Cañas R., De la Torre F., Pascual M., Avilam C., Cánovas F. (2016). Nitrogen economy and nitrogen environmental interactions in conifers. Agronomy.

[66] Qi M. (2007). Achievements, problems and countermeasures of clonal selection and breeding of Chinese Fir in China. World For. Res..

[67] Mantel S., Dondeyne S., Deckers S. (2014). World Reference Base for Soil Resources (WRB). Encyclopedia of Soils in the Environment.

[68] Condit R. (1998). Tropical Forest Census Plots: Methods and Results from Barro Colorado Lsland, Panama and a Comparison with Other Plots.

[69] Zhu Y., Comita L.S., Hubbell S.P., Ma K. (2015). Conspecific and phylogenetic density-dep endent survival differs across life stages in a tropical forest. J. Ecol..

